# Assessment of cognitive safety in clinical drug development

**DOI:** 10.1016/j.drudis.2015.11.003

**Published:** 2015-11-22

**Authors:** Jonathan P. Roiser, Pradeep J. Nathan, Adrian P. Mander, Gabriel Adusei, Kenton H. Zavitz, Andrew D. Blackwell

**Affiliations:** 1 Institute of Cognitive Neuroscience, University College London, London, UK; 2 Department of Psychiatry, University of Cambridge, Cambridge, UK; 3 MRC Biostatistics Unit, University of Cambridge, Cambridge, UK; 4 Cambridge Cognition Limited, Bottisham, Cambridge, UK; 5 Neuroscience Center of Excellence, inVentiv Health, Maidenhead, UK; 6 School of Psychological Sciences, Monash University, Clayton, Australia

## Abstract

Cognitive impairment is increasingly recognised as an important potential adverse effect of medication. However, many drug development programmes do not incorporate sensitive cognitive measurements. Here, we review the rationale for cognitive safety assessment, and explain several basic methodological principles for measuring cognition during clinical drug development, including study design and statistical analysis, from Phase I through to postmarketing. The crucial issue of how cognition should be assessed is emphasized, especially the sensitivity of measurement. We also consider how best to interpret the magnitude of any identified effects, including comparison with benchmarks. We conclude by discussing strategies for the effective communication of cognitive risks.

## Introduction

Assessing cognitive safety, in other words the impact of clinical treatments on the ability to perceive, process, understand, and store information, make decisions and produce appropriate responses, is an issue whose importance is increasingly recognised by the pharmaceutical industry, regulators, clinicians, and the public. In some cases (e.g., first-generation antihistamines), marked cognitive-impairing effects were established many years ago, and warnings relating to possible sedation routinely appear on labelling [1]. More recently, there has been widespread concern about possible adverse effects of several commonly used drugs that, although not necessarily causing marked sedation, are likely to have important cognitive effects. For example, several epidemiological cohort studies report that impaired cognitive function is associated with medications that have anticholinergic activity, particularly when taken in combination [2]. One large study in older patients reported consistently impaired scores on the Mini Mental State Examination (MMSE: a dementia rating scale) in those using medication with definite anticholinergic activity, after adjusting for several confounders [3]. Other studies reported that the use of anticholinergics is associated with increased risk of mild cognitive impairment (MCI) [4], and even with dementia in a dose-dependent fashion [5,6].

The above results present a clear cause for concern in older people, but it is also important to consider the potential impact of cognitive impairment on wellbeing and everyday function in younger populations. In the workplace, medication-induced cognitive impairment could result in reduced productivity, or ‘presenteeism’, and could be dangerous for those who drive or operate machinery as part of their jobs. At school or university, cognitive impairment could prevent students from fulfilling their academic potential, with implications for future competitiveness in the jobs market.

Medication-induced cognitive impairment also raises cause for concern outside work or study contexts: everyday tasks are likely to be adversely affected. Driving is one of the best studied of these, and initiatives such as the DRiving Under the Influence of Drugs, alcohol and medicines project (DRUID [7]) have highlighted classes of medicine that are likely to induce cognitive impairment, based on a review of the pharmacological, epidemiological, and experimental psychopharmacological literature. Laboratory studies examining alcohol administration (which is well known to impair driving performance and, therefore, can act as a standard reference [8]), have highlighted several core cognitive processes that, if disrupted by a drug, are likely to impair driving ability and warrant further investigation [9]. Additionally, there is an extensive literature on the effects of common medicines on actual driving ability, measured using either a specially instrumented vehicle on a public highway in normal traffic or a driving simulator [10], which has identified several drug classes that are likely to increase the risk of road traffic accidents [11].

Whereas driving ability has been particularly well studied, and warnings not to drive or operate machinery have appeared on medication labelling for decades, these are not the only important aspects of everyday function that are likely to be affected if cognition is disrupted. This issue is well recognised in the literature on cognitive decline in older individuals, which focuses on activities of daily living (ADL) [12]. Use of communication devices (and technology more generally), managing finances, cooking meals, shopping, navigation, and housework can all be adversely affected by medication-induced cognitive impairment. Everyday tasks that are more cognitively demanding (often termed ‘instrumental’ ADLs), such as passing on a message to another person, finding the way in an unfamiliar place, or taking part in a conversation, are particularly affected when cognitive ability is disrupted [13], and are frequently impaired early in the course of cognitive decline. Such impairment of everyday function substantially reduces quality of life in patients and is a major contributor to burden on caregivers [14]. The association between cognitive impairment and ADL is also pronounced in younger individuals with mental illness, for example schizophrenia [15] and bipolar disorder [16]. Indeed, several studies have found that in those recovering from schizophrenia, cognitive performance predicts resumption of normal function (such as the ability to live independently, participate in leisure activities, and return to the workplace) better than symptoms [17].

Cognitive impairment clearly represents an important possible adverse effect of medication. Regulators are likely to demand, and consumers have the right to be informed about, possible cognitive risks. However, the degree to which many medications influence cognitive function remains unknown. In this article, we outline experimental approaches to determining whether a drug impacts cognition, discussing: (i) the rationale for assessing cognition during clinical development; (ii) drug classes likely to affect cognition; (iii) study design, populations and analysis; and (iv) the importance of using sensitive and comprehensive measurements. We conclude by considering how to interpret any effects detected, and strategies for communicating the potential implications of any findings to regulators and consumers.

## Why is it important to assess cognition during clinical drug development?

Assessment of safety and tolerability (e.g., cardiovascular effects, changes in liver enzymes, neurological events, etc.) is a crucial component of early-phase clinical studies. A decision to progress a candidate compound to later phases depends on the outcome of these studies. Many central nervous system (CNS) and non-CNS compounds have the potential to affect cognitive ability detrimentally, and the risks have been highlighted for particular drug classes [18–21]. Drugs can have multiple pharmacological effects, some of which are desirable (related to mechanism and/or target of interest) and some undesirable (‘off-target’, or other pharmacology leading to adverse effects, such as cognitive dysfunction). If a drug impairs cognition, this might be related to off-target pharmacological effects known to impair cognition (e.g., blockade of muscarinic, histaminergic, or beta adrenergic receptors). Given the greater awareness of potential cognitive impairment by regulators [e.g., US Food and Drug Administration (FDA) advice on statin risks: http://www.fda.gov/Drugs/DrugSafety/ucm293101.htm], as well as the general public, changes in cognition are increasingly assessed using objective measures during clinical development to examine the potential short- and long-term cognitive risks. As such, assessment of cognitive function can be an integral part of decision making during clinical development.

**FIGURE 1:**
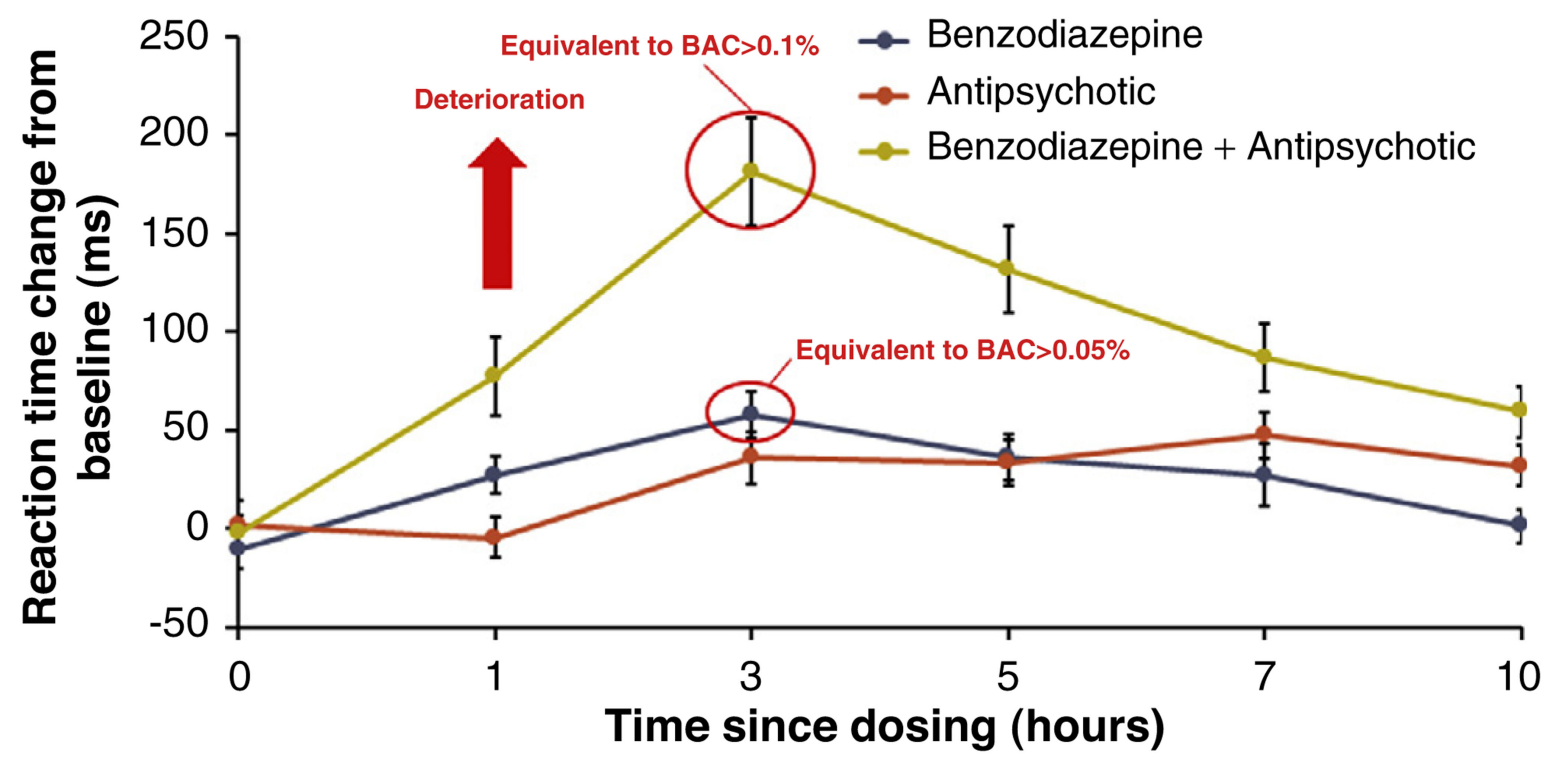
The effect of an antipsychotic and a benzodiazepine drug on reaction times when administered in combination is greater than the sum of each separately, indicating a drug–drug interaction. At the time of maximum impairment (3 h), responses on a choice reaction time test were slowed by approximately 30 ms by the antipsychotic, approximately 50 ms by the benzodiazepine [greater than the effect of blood alcohol concentration (BAC) of 0.05 g/dL, the legal driving limit in many countries], but by approximately 175 ms when these were administered in combination (greater than the effect of BAC of 0.1 g/dL). If driving at a speed of 100 kph (60 mph), a slowing of response of approximately 175 ms is equivalent to an increased stopping distance of approximately 4.9 m (approximately 15 ft), the length of a large sedan car. *Source*: Unpublished data, kindly provided by Otsuka Pharmaceutical Co., Ltd.

Information on the cognitive profile of a compound could be informative for several reasons and can aid decision making during clinical development. For example, adverse effects on cognitive function might be important for: (i) determining dose–response relationships and selecting safe doses for later phase development (including the need for titration); (ii) differentiation from competitor drugs in relation to cognitive safety; (iii) detecting off-target pharmacological effects (i.e., other pharmacology unrelated to the target of interest); and (iv) assessing the risk:benefit ratio in relation to the target indication. In addition, it might also be worthwhile to continue to assess cognitive safety following approval and marketing through monitoring of cognitive function as part of routine pharmacovigilance, which is increasingly feasible through the use of internet- or app-based assessments. Longer-term monitoring of cognition might be particularly useful in detecting effects of drug–drug interactions on cognitive function (Fig. 1), especially in individuals with multiple co-morbidities (for whom polypharmacy is the norm), because such patients are usually excluded from clinical trials.

### Regulatory expectations and compliance

The importance of assessing cognition during clinical development is outlined in several FDA guidance documents. For example, in guidance document UCM126958, published by both the Center for Drug Evaluation and Research and the Center for Biologics Evaluation and Research, the FDA highlights the fact that certain types of adverse effect are likely to go undetected if specific, sensitive measurements are not used [22]. This document recommends that, when a drug has the potential for such effects, additional testing or specific assessments will be required. As an example, it states: ‘for a new drug with recognised CNS effects (especially sedating effects), sponsors should conduct an assessment of cognitive function, motor skills, and mood’.

More recent FDA guidance provides an even clearer expectation of cognitive safety assessment during clinical development. The draft document UCM430374 discusses the evaluation of drug effects on the ability to operate a motor vehicle (http://www.fda.gov/downloads/Drugs/GuidanceComplianceRegulatoryInformation/Guidances/UCM430374.pdf). It states: ‘Beginning with first-in-human studies, all drugs, including drugs intended for non-CNS indications, should be evaluated for adverse effects on the CNS… The occurrence of adverse CNS events in even a small number of phase 1 subjects can indicate the need for more focused studies of CNS effects. Early testing for CNS effects should generally emphasize sensitivity over specificity … measures of reaction time, divided attention, selective attention, and memory may be appropriate’. These regulatory expectations warrant the use of specific, targeted, and sensitive cognitive safety assessments, because routine monitoring will at best underestimate adverse effects and at worst fail to detect them completely.

The extent to which better monitoring of cognitive outcomes during drug development can lead to better regulated medicines is increasingly recognised as important in the wider regulatory context. Although assessments establishing the cognitive effects of drugs have been applied in pharmacological studies since the late 1960s [23], the use of objective tests (as opposed to self-report measures) has typically not been a requirement of regulatory bodies. Additionally, those early studies that did use objective cognitive assessments during the drug development process often focused on psychomotor function or processing speed, and did not examine higher-order cognitive processes, such as executive function, social cognition, or specific components of memory. Consequently, objective cognitive assessment has not been conducted as consistently or comprehensively as other types of safety assessment during clinical development, resulting in uncertainty about the cognitive impact of many commonly used medicines [24]. Therefore, it is of clear regulatory interest to incorporate such exercises into ongoing safety monitoring for currently marketed drugs.

## Compounds likely to have a negative impact on cognition

### CNS disorder drugs

Broadly speaking, any drug that is CNS penetrant (i.e., crosses the blood–brain barrier) can influence cognition through effects on neurotransmitter systems, such as dopamine, acetylcholine, noradrenaline, glutamate, GABA, histamine, adenosine, and serotonin. More specifically, compounds that boost the function of specific neurotransmitter systems (e.g., agonists, reuptake inhibitors, or releasers), or block transmission in these systems (e.g., antagonists at postsynaptic receptors) might influence cognition. This includes many compounds developed for neurological disorders, such as epilepsy (i.e., anticonvulsants) and chronic pain, as well as neuropsychiatric disorders (reviewed in [2,25–30]). The effects of compounds on cognitive function might be nonlinear; for example, following an inverted-U function, as observed for drugs affecting the dopamine system, with either too little or too much transmission impairing cognition [31]. Such effects are not simply of academic interest; they are highly relevant for drugs in development for Parkinson’s disease (i.e., modulators of dopamine transmission) because it is likely that, although motor symptoms might be improved, there could be detrimental effects on specific cognitive processes [32,33]. In the specific case of drugs that stimulate dopamine D2/D3 receptors, this might include unwanted influences on reward processing and/or impulsivity [34], which are warning flags for abuse liability.

### Non-CNS disorder drugs

Whereas the potential for drugs developed for CNS disorders to impact cognitive function detrimentally is clear, there are also many classes of compound developed for non-CNS disorders that confer a risk of cognitive impairment. Known examples include those developed for cardiovascular disorders [18,21], obesity [19], oncology [35,36], genitourinary disorders (e.g., overactive bladder [37,38]), and allergies [39]. In many cases, it is unclear what processes are responsible for the deleterious effects on cognition. However, possible mechanisms include: (i) indirect effects on central neurotransmission; (ii) effects on metabolic function (e.g., glucose, hormones); (iii) effects on the immune system (e.g., cytokines), which communicates extensively with the CNS; and (iv) other adverse events (e.g., nausea or pain). There is also growing evidence that the integrity and permeability of the blood–brain barrier can be compromised by many common medical conditions, including systemic diseases (e.g., diabetes, hypertension, and hypercholesterolemia), inflammatory conditions (multiple sclerosis), neurodegenerative diseases (e.g., Alzheimer’s disease and Parkinson’s disease), infections such HIV, stroke, traumatic brain injury, and brain tumours [40–42]. Furthermore, the function of the blood–brain barrier can be altered by certain medications, environmental toxins, and the ageing process itself [41]. Therefore, there exists the potential for many new and commonly used drugs to gain access to the brain and have an unanticipated impact on cognitive function, in both clinical trials and real-world settings.

## Study design and analysis

Epidemiological cohort studies can provide suggestive evidence that a drug might influence cognition, and often benefit from large sample sizes resulting in robust statistical inference. However, experimental clinical studies, in particular double-blind, randomised controlled trials (RCTs), should be considered the gold standard when assessing cognitive safety. Although epidemiological studies enjoy the benefits of population samples, they are purely observational and, therefore, can be subject to confounding, by measured or unmeasured variables, meaning that causal inferences can be difficult. Drawbacks of RCTs include that they are time consuming to conduct and costly, and that the sample tested might not be completely representative of the population that will eventually take the drug. However, ultimately, they provide the best quality of evidence addressing the question of cognitive safety and, therefore, represent high value for money.

### Phase I

In Phase I trials, cognitive impairment can be considered either at the level of the individual subject or across groups, alongside other commonly assessed adverse events. Inferential statistics might or might not be conducted, but examining patterns in cognitive data will nonetheless be informative. Cognitive assessments typically produce continuous measurements, and large databases of normative scores exist for many commercially available tests; meaning that the normal range in performance (given a subject’s age and educational level) can be calculated and used to determine the likely importance of any observed fluctuations. The standard approach to cognitive assessment in clinical trials is to take measurements both after drug administration and at baseline (usually the point of randomisation); including the latter in statistical analyses improves sensitivity because natural interindividual variability in performance can be accounted for.

One possible approach to declaring a cognitive adverse event would be if a subject scored within the normal range at baseline, but following drug administration performance dropped below a threshold based on the reliable change (RC) index, a metric derived from the test–retest reliability of a measure [43]. For cognitive measures, the RC index might need to be adjusted for practice effects (because people’s scores naturally improve following repeated exposure to the same test). Such a categorical approach might be particularly useful in small Phase I studies, but inferential statistics would not usually be performed. Isolated incidents of poor cognitive performance would not necessarily preclude further drug development, although they might flag up areas for consideration in later phases.

Alternatively or additionally, cognitive scores before and after drug administration can be averaged within dose groups and compared statistically with an active or nonactive comparator. However, often such comparisons will be limited by poor statistical sensitivity (see below), even after accounting for baseline performance, because of the low numbers of subjects typically included in Phase I studies. As such, results from Phase I cognitive assessments would usually not be considered conclusive. Depending on the target indication, if consistent cognitive safety signals were observed across most or all subjects, this might form part of the basis of a decision not to progress a compound through the development pipeline, because of the possibility that the degree of impairment induced might outweigh the potential clinical benefit. Therefore, checking cognitive effects early in development could contribute meaningful information to risk management and go/no-go decisions.

### Phases II and III

Cognitive safety is also considered at the group level beyond Phase I, averaging observations over dozens or hundreds of individuals per study arm. However, assessing cognitive safety raises some important design and statistical challenges.

#### Superiority designs

By its nature, demonstrating cognitive safety requires asking a very different question to that addressed in standard ‘superiority’ trials, where the aim is to determine whether a drug performs better than some comparator (e.g., placebo, a lower dose of the same compound, or an existing in-market drug). A desirable outcome in superiority trials is to show a difference between the conditions. The standard (Neyman–Pearson, or frequentist) statistical framework derives a *P* value: the probability that a pattern of results at least as extreme as that observed would occur under the null hypothesis (*H*_0_: that the drug under study and the comparator have the same effect). If the *P* value falls below a prespecified value (alpha, the tolerance for the frequency of false positives; conventionally set at 5%), the result is declared significant and *H*_0_ is taken to be rejected. In the frequentist framework, the interpretation of a significant *P* value in the context of a superiority trial as supporting a rejection of the null is logically unambiguous (at a given false positive tolerance level).

By contrast, demonstrating ‘cognitive safety’ requires asking a different question: in this case, a desirable result will often be to conclude that there is no difference between a drug and some comparator. Adopting a strict interpretation of cognitive safety, we might wish to ask whether cognitive function while taking a drug is no worse than if the drug had not been administered. Using a standard placebo-controlled RCT, failing to show a significant difference between an active treatment and a nonactive comparator is not, by itself, sufficient to demonstrate cognitive safety. This is because failing to show a significant difference can be an ambiguous result in the frequentist statistical framework: absence of evidence is not necessarily evidence of absence. A nonsignificant *P* value can leave the investigator trapped in the logical straightjacket of a triple negative: a failure (1), to reject (2), the null (3); a position from which it can be difficult to draw any meaningful conclusions at all. Therefore, *P* values cannot, by themselves, provide evidence in favour of *H*_0_, and the notion of a result being ‘highly nonsignificant’ is logically meaningless [44].

A nonsignificant result could reflect a true negative; in other words, there is genuinely no difference in the cognitive impact of the drug and the comparator. However, nonsignificance could also occur even when a difference truly exists, because of low sensitivity (i.e., a false negative). A common reason for false negatives is low statistical power; that is, not including enough subjects to detect an effect of a magnitude considered clinically important. This must be addressed before the study with a power calculation. If a minimally important effect size can be specified in advance (which can itself pose a challenge), then the number of subjects required to reject the null hypothesis (at given tolerance levels for false negatives and positives) can be calculated. To provide a credible demonstration that two conditions do not differ, the tolerance for false negatives (Type II errors) needs to be controlled at a sufficiently low rate, just as the tolerance level for false positives (Type I errors) needs to be controlled in superiority designs, perhaps also at 5%. This requires large samples. For example, with the false positive and false negative rates both set to 5%, testing for an effect of standardised mean difference (SMD, Cohen’s *d*) = 0.3 (equivalent to a typical antidepressant effect size [45]) would require nearly 250 individuals in each arm using a one-tailed statistic (300 if a two-tailed test were planned, additionally allowing for the possibility that the drug under study improves cognition). Assuming that the sensitivity of measurement can be assured (see section on ‘Measurement of cognition’ below) a nonsignificant result arising from an adequately powered superiority RCT could be interpreted as indicating that the effect of the drug in question on cognition is no greater, relative to the comparator, than the effect size specified in the power calculation.

#### Noninferiority designs

Another option, adopting a slightly different interpretation of cognitive safety, would be to use a ‘noninferiority’ design [46], in which the drug under study is compared against an active comparator. Noninferiority trials are often used to establish efficacy in situations when administering placebo would be considered unethical, for example when an established treatment is clearly effective and to administer placebo would expose patients to serious risk. Such designs allow the conclusion that a new drug is ‘no less effective’ (within some margin; see below) than an existing compound with established efficacy. The logic in the context of a study on cognitive safety is slightly different. If a comparator drug has a clearly detrimental influence on cognition, with a well-established effect size (which might be considered acceptably low), it can act as a benchmark for the drug under study. If noninferiority can be demonstrated, it can be concluded that the drug under study is at least ‘no more detrimental’ (within some margin) than the active comparator.

In such designs, it is necessary to define a noninferiority margin (M): the extent to which the drug under study could perform worse than the active control but still be considered similarly effective (or harmful, in the case of safety). The starting point for specifying M is the expected effect of the active comparator relative to placebo, usually known before the study commences from existing data. FDA guidance states that M can be ‘no larger than the entire effect that [the active control] is presumed to have had [relative to a placebo condition, had it been included]’ (http://www.fda.gov/downloads/Drugs/Guidances/UCM202140.pdf). Typically, a smaller (i.e., more conservative) estimate of M than this will be used. For example, it might be decided that M should lie within some percentage of the effect of the active control; alternatively or additionally, confidence intervals from historical data and/or clinical judgment might be used. To demonstrate noninferiority, the 95% confidence interval derived from the contrast of the drug under study against the active comparator should not overlap with M.

Although specification of M poses a challenge in conducting and interpreting such studies, it is arguably no greater than the challenge in specifying a minimally interesting effect size when calculating statistical power in a superiority design. However, if a conservative (i.e., small) M is adopted, large sample sizes might be required. As well as the potential to demonstrate noninferiority, a further possibility is that cognitive performance is actually significantly better following administration of the drug under study than the active comparator (similar to a superiority design). Therefore, it is useful to allow for the additional possibility of subsequent testing for superiority over the active comparator (assuming that noninferiority has already been demonstrated), because this would permit an unambiguous demonstration of (relative) cognitive safety if statistical significance were achieved.

#### Hybrid designs

The usual aim in noninferiority studies is to demonstrate that the drug under study performs no worse that the active comparator, within the margin, M. This can be achieved using a two-arm design (http://www.fda.gov/downloads/Drugs/Guidances/UCM202140.pdf). Such a design assumes that the M can be specified confidently and appropriately on the basis of historical data (the ‘constancy’ assumption); however, this is not always the case. For example, data might not be available examining the contrast of the active comparator against placebo on a particular cognitive measure, or in a specific population. To facilitate the specification of M in such cases, it might be useful to incorporate an additional nonactive (i.e., placebo) comparator condition, allowing for an empirical estimation.

A convincing result supporting cognitive safety from such a design would be to demonstrate two effects: (i) the active comparator impairs cognition significantly relative to placebo. This demonstrates study (and measurement) sensitivity. The placebo data can then also be used to inform the estimate of M; and (ii) after M has been specified, the drug under study can then be tested for noninferiority relative to the active comparator. If both (i) and (ii) yield significant results, this design enables the conclusion of noninferiority using an empirically informed M. However, if (i) fails to achieve significance, this indicates poor study sensitivity, possibly because the sample size was not sufficiently large, or the measurement was not sufficiently sensitive.

### Further design and analysis considerations

#### Bayesian analysis

An alternative statistical approach would be to use a Bayesian procedure, which takes a complementary perspective to the standard frequentist framework to make inference. Instead of computing the probability of the observed data, conditioned on *H*_0_, the Bayesian approach computes the probability of the hypothesis in question, conditioned on the observed data. Note that the term ‘probability’ in the Bayesian framework is applied to hypotheses, not data, so strictly refers to subjective, not objective probability; that is, confidence: how certain one is that a particular hypothesis is correct. Bayesian inference also requires the specification of ‘priors’; that is, pre-study predictions about experimental data (although if these are specified weakly, they have little influence on the conclusions drawn). The observed data are combined with the priors through application of Bayes’ rule to create a ‘posterior’ estimate, together with a ‘credibility interval’ (the Bayesian equivalent of a confidence interval), which provides the basis for inference.

The particular value of Bayesian statistics in the assessment of cognitive safety is that it allows firm conclusions to be drawn supporting the null hypothesis. Bayesian statistics can also incorporate existing data in priors (e.g., the performance of individuals administered placebo, perhaps established through previous work), which might allow for a smaller number of subjects to be tested. Finally, Bayesian inference can allow strong conclusions to be drawn on the basis of relatively small datasets (see [47] for a discussion).

#### Missing data

Missing data are important to consider in any trial, but particularly in the context of cognitive safety, regardless of the design or analysis used. If a drug is cognitively impairing, then this effect itself could conceivably increase the chance that participants will drop out of the trial, or fail to attend or complete a testing session. If this occurs, and the probability that a data point is missing depends on this unobserved value, then the data are ‘missing not at random’ (MNAR), which could bias subsequent analysis towards a null effect if a per-protocol analysis is used. In other words, a per-protocol analysis could fail to detect a genuine cognitive safety signal (false negative) because the subjects who were most cognitively impaired by the drug were not tested. However, intent-to-treat analysis with last-observation-carried-forward (LOCF) is also not an advisable strategy in cognitive safety trials. It will be biased towards showing no difference when data are MNAR, because LOCF will tend to underestimate effects. This strategy results in a more conservative analysis in the context of testing for efficacy, but a more liberal one in the context of cognitive safety. Thus, correcting for MNAR data in the context of cognitive safety is not trivial and careful consideration needs to be given to the method used to account for it.

## Study population and time period

Important questions for any trial are what study populations should be considered and over what time period. It is likely that initial cognitive safety studies will be conducted in healthy volunteers, which should explore a range of doses over a variety of timescales. Single dose (acute) or repeated dose (chronic) both need to be considered, depending on whether the drug in question is intended for use over an extended period of time.

However, even if studies in healthy volunteers provide results consistent with cognitive safety, it would still be important to test the cognitive effects of a drug in patient populations. Indeed, it is possible that cognition might be adversely affected by a given drug in healthy volunteers, while in the target patient population effects on cognition are minor (because the concomitant alleviation of symptoms can have a positive effect on cognitive function). For example, donepezil is known to impair cognition in healthy volunteers [48], but nonetheless is approved as a treatment for dementia. It is also possible that the cognitive effects of a drug might interact with developmental stage in younger populations (with potentially important implications for academic attainment if patients are still studying), or neurodegeneration in older or cognitively vulnerable individuals. Finally, cognitive effects of drugs might occur cumulatively, as shown by the example of anticholinergic load outlined in the introduction; therefore, additive or interactive effects with other drugs might be important to consider (Fig. 1). This has important implications for real-world clinical practice, because clinicians assessing risk and prescribing need to consider other drugs that their patients might be taking.

## Measurement of cognition

**FIGURE 2:**
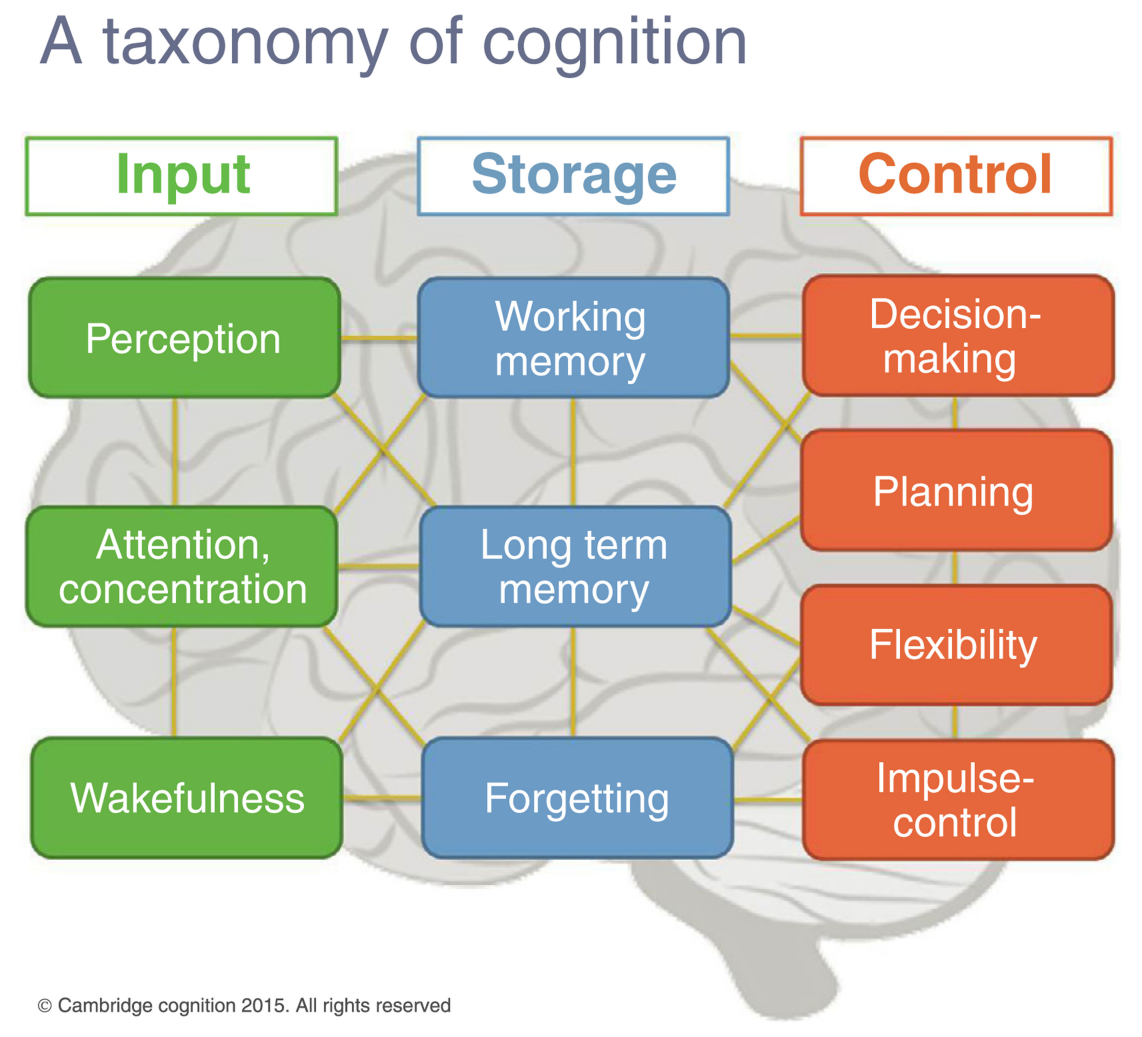
Commonly assessed components of cognition, broadly split into the domains of input, storage, and control. *Source*: Reproduced, with permission, from Cambridge Cognition.

Broadly speaking, cognitive assessments measure an individual’s information processing capacity, including concentration, storage, and control (Fig. 2). The sensitivity and comprehensiveness of measurement is a crucial consideration in cognitive safety studies, especially in the common situation that inference will be based on statistically nonsignificant results. Even in an otherwise adequately powered study, a misleading nonsignificant result could arise if an insensitive instrument was used to assess cognition, or was applied incorrectly. Therefore, it is important to utilise sensitive, standardised cognitive assessments that have demonstrated sensitivity to cognitive impairment. The measurements used should also have adequate test–retest reliability and low practice effects to maximise sensitivity. A common strategy has been to assess cognitive impairment through self-report questionnaires [19]. However, in many cases, individuals will not have good insight into their own cognitive ability, so this might be insufficient to exclude all but the most pronounced effects on cognition.

Choosing appropriate tests for the study population in question is another important aspect of cognitive safety. If a test is too easy or too difficult then sensitivity will be compromised because of measurement boundary effects. For example, a test such as the MMSE (routinely used in the detection of dementia) will not be suitable to detect effects in young healthy subjects. Ideally, the test used should incorporate different levels of difficulty, which might be adjusted adaptively, furnishing sensitivity to detect cognitive impairment across the range of ability.

Until the late 1960s, cognitive assessments almost exclusively used traditional paper-and-pencil measures, many of which were based on tests originally developed for the US Army to screen recruits. These tests are still used as part of standardised neuropsychological assessment in clinical settings. However, from the 1970s onwards, investigators began to use computerised testing to assess the cognitive effects of drugs [23], including automated versions of earlier paper-and-pencil tests [49] as well as new tests intended to assess specific domains of cognitive function [24]. The advent of automated computerised testing saw several improvements in the measurement of cognition. These include: increased precision of measurement, especially in relation to response speed; standardised timing of presentation of stimuli; greatly reduced potential for administrator errors or bias; improved portability; and increased efficiency (because data do not require digitisation before analysis). Automated computerised tests also have the advantage that they can be administered by less specialist staff, at substantially lower cost. However, despite these advantages, such cognitive assessments have not been used routinely as safety assessments during clinical development.

The term ‘cognition’ covers a range of processes, including (among others) perception, working memory (maintaining information ‘on-line’), episodic memory, sustained attention, decision making, and motor performance. Therefore, it is desirable to incorporate multiple tests into any assessment (although investigators might decide to focus on cognitive functions likely to be affected by the particular mechanism of action of the drug). For example, if memory is not assessed at all during cognitive assessment, then the study cannot draw any conclusions about the effects of the drug on this process, which might obscure potential implications for everyday function. Importantly, it is possible that a drug could impair certain cognitive processes while leaving others intact, resulting in a profile of cognitive impairment. The use of a range of cognitive measures is also an important statistical consideration, because it will increase the number of comparisons made and thereby the false positive rate. One possible solution is to compute a composite measure across tests, but this approach is only valid when the tests all assess a common process.

## Interpreting a cognitive safety signal

A challenge associated with the assessment of cognition is the interpretation of any signals observed. In addition to the statistical methods discussed above, it is important to communicate the magnitude of any detrimental effects in context. While statistically significant impairments might be detected, it does not automatically follow that these will be clinically meaningful.

Several possible approaches can be used to decide whether an effect is clinically meaningful. One would be to determine whether the estimated effect size of the impairment falls within conventional limits for small, medium or large effects. Following Cohen’s convention [50], an effect size (SMD) of <0.3 might be considered small, and might not be clinically meaningful. However, unlike efficacy studies, in which one might feel comfortable in accepting effect sizes of SMD < 0.3 as not clinically meaningful, a more risk-averse perspective might be warranted when considering cognitive safety. Hence, the criterion for nonclinical relevance might need to be more stringent; for example, an effect size of SMD < 0.2 or lower. However, the precise level set would also need to consider the risk–benefit profile for the drug under study in relation to the target indication. When incorporating multiple tests to assess cognition across cognitive domains, the domain or combination of domains affected might further contribute to the evaluation of whether an effect is a clinically meaningful. The criterion should be set proportionately more strictly for indications where the risks of treatment might outweigh the benefits (e.g., developmental conditions).

One could also consider the effect size detected relative to that observed in neuropsychiatric disorders; for example, impairments of SMD = 1.5 (or higher) occur in cognitive disorders, such as dementia or MCI [51]. Schizophrenia and depression, in which cognitive impairments are considered core symptoms, are associated with impairments of around SMD = 1 and SMD = 0.5, respectively [52,53]. Typically, SMDs of 0.65 or greater (equivalent to around a ten-point drop in performance IQ) would be considered clinically relevant; hence, an impairment of this magnitude would indicate a clinically significant safety signal.

**FIGURE 3:**
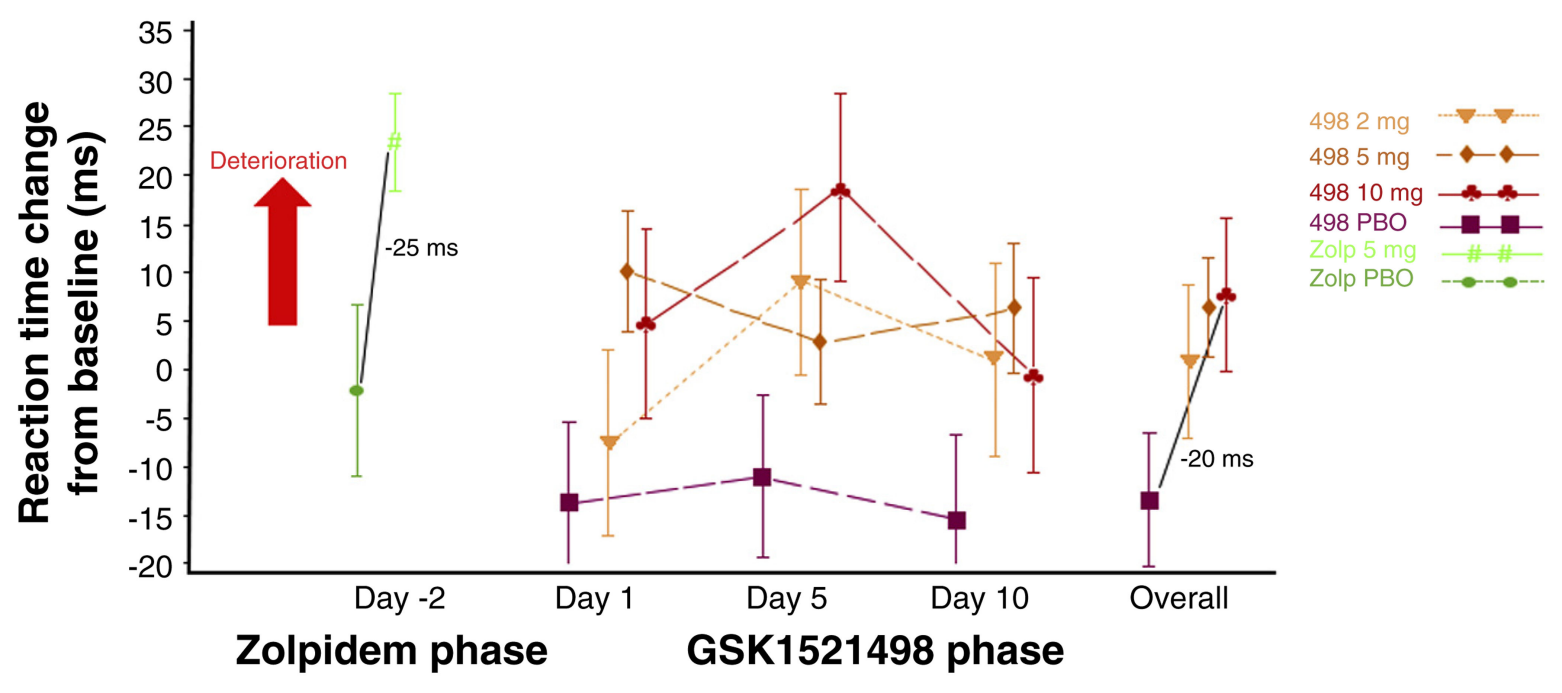
Results from a Phase I study of the comparative effect of 5-mg zolpidem (benzodiazepine with sedative properties: left side of figure) and GSK1521498 (µ-opioid inverse agonist in development for obesity and/or addiction: middle–right side of figure) on reaction times during a test of attention. Zolpidem (Zolp) slowed responses, relative to placebo, to approximately the same degree as previously shown at a blood alcohol concentration of 0.05 g/dL (approximately 25 ms, green points [55]). Even at the highest dose tested, the average impairment caused by GSK1521498 (approximately 20 ms, red/orange points) was lower than that caused by 5-mg zolpidem, consistent with a relatively low cognitive risk and supporting the continued clinical development of the compound. Abbreviation: PBO, placebo. *Source*: Reproduced, with permission, from [54].

An alternative approach is to benchmark any observed cognitive safety signals against socially acceptable levels of impairment. In this regard, considering the impairment elicited by alcohol at the legal driving limit (i.e., 0.05–0.07 g/dL in most countries), overnight sleep deprivation, or healthy ageing might be useful. For example, this approach was used in assessing the safety risk of a novel compound, GSK1521498, in Phase I development for obesity/addiction [54] (Fig. 3). In this study, a 5-mg dose of the sedative drug zolpidem (active comparator), which causes a decrement in reaction time during sustained concentration similar to the minimum impairment elicited by alcohol at 0.05 g/dL (approximately 25 ms, SMD approximately 0.7) [55], was used to examine the cognitive risk associated with GSK1521498 in a comparative manner. As expected, 5-mg zolpidem caused a significant reaction time impairment (approximately 25 ms), confirming the sensitivity of the cognitive measurement and study design. However, most effects of GSK1521498 on cognition were nonsignificant relative to placebo, and those impairments observed were numerically smaller than that caused by zolpidem (approximately 20 ms averaged across three time points at the highest dose), indicating a relatively low cognitive safety risk and supporting the continued clinical development of the compound.

## Concluding remarks

Here, we have reviewed the rationale for examining cognitive safety during clinical development, possible study designs and analytic approaches, considerations relating to measurement sensitivity, and strategies for interpreting and communicating any cognitive safety signals. This field is still at an early stage, and precisely what designs should be adopted, what outcome measures should be used, and what statistical approaches are most appropriate will vary depending on the drug in question and the indication. Although we have proposed some approaches that might be useful and made some initial recommendations, there is as yet no consensus on the best way to demonstrate cognitive safety, or even how this term should be interpreted. Ultimately, prescribing medication is about weighing up the potential for risks and benefits. Even if a drug is shown to induce some cognitive impairment, it might still be beneficial to prescribe it; but pharmaceutical companies, regulators, clinicians, and patients need to understand the possible cognitive risks, and their implications for everyday function.
